# No Influence of the Fat Mass and Obesity-Associated Gene rs9939609 Single Nucleotide Polymorphism on Blood Lipids in Young Males

**DOI:** 10.3390/nu12123857

**Published:** 2020-12-17

**Authors:** James L. Dorling, Alice E. Thackray, James A. King, Andrea Pucci, Fernanda R. Goltz, Rachel L. Batterham, David J. Stensel

**Affiliations:** 1 National Centre for Sport and Exercise Medicine, School of Sport, Exercise and Health Sciences, Loughborough University, Loughborough LE11 3TU, UK; james.dorling@pbrc.edu (J.L.D.); A.E.Thackray@lboro.ac.uk (A.E.T.); J.A.King@lboro.ac.uk (J.A.K.); Fernanda.ReistenbachGoltz@rdsi.nestle.com (F.R.G.); 2 National Institute for Health Research (NIHR) Leicester Biomedical Research Centre, University Hospitals of Leicester, National Health Service (NHS) Trust and the University of Leicester, Leicester LE1 5WW, UK; 3 Centre for Obesity Research, University College London, London WC1E 6JF, UK; andrea.pucci@uclh.nhs.uk; 4 University College London Hospitals Bariatric Centre for Weight Management and Metabolic Surgery, Ground Floor West Wing, 250 Euston Road, London NW1 2PG, UK; 5 Nestlé Product Technology Centre, Lange Str. 21, 78224 Singen, Germany

**Keywords:** cholesterol, genetics, triacylglycerol, insulin resistance, obesity

## Abstract

The fat mass and obesity-associated gene (*FTO*) rs9939609 A-allele is linked to obesity and dyslipidemia, yet the independent influence of this polymorphism on blood lipids remains equivocal. We examined the influence of the *FTO* rs9939609 polymorphism on fasting and postprandial blood lipids in individuals homozygous for the risk A-allele or T-allele with similar anthropometric and demographic characteristics. 12 AA and 12 TT males consumed a standardized meal after fasting overnight. Blood samples were collected at baseline (−1.5 h), before the meal (0 h), and for five hours postprandially to measure lipid, glucose, and insulin concentrations. Time-averaged total area under the curve (TAUC) values (0–5 h) were calculated and compared between genotypes. Fasting triacylglycerol (TG), high-density lipoprotein cholesterol, low-density lipoprotein cholesterol, total cholesterol, non-esterified fatty acid (NEFA), glucose, and insulin concentrations were similar between groups (*p* ≥ 0.293). TAUC for TG was similar in AAs and TTs (95% confidence interval (CI) −0.52 to 0.31 mmol/L/h; *p* = 0.606). Likewise, TAUC values were similar for NEFA (95% CI −0.04 to 0.03 mmol/L/h; *p* = 0.734), glucose (95% CI −0.41 to 0.44 mmol/L/h; *p* = 0.951), and insulin (95% CI −6.87 to 2.83 pmol/L/h; *p* = 0.395). Blood lipids are not influenced by the *FTO* rs9939609 polymorphism, suggesting the *FTO*-dyslipidemia link is mediated by adiposity and weight management is important in preventing *FTO*-related lipid variations.

## 1. Introduction

At the fat-mass and obesity-associated gene (*FTO*) rs9939609 single nucleotide polymorphism (SNP), carriers of the obesity-risk A-allele present a higher risk of obesity compared to homozygous carriers of the T-allele in Europeans [1,2], primarily because of elevations in appetite and energy intake [3,4]. Further work also reports an increased propensity for cardiovascular disease (CVD) in carriers of the *FTO* rs9939609 A-allele [5,6]. Doney et al. showed that carriers of the *FTO* rs9939609 A-allele had lower high-density lipoprotein cholesterol (HDL) and higher random triacylglycerol (TG) concentrations than non-carriers, indicating that the *FTO*-CVD relationship may be explained by differences in blood lipids [7]. Nonetheless, others have shown no influence of the *FTO* A-allele on fasting blood lipid profiles [8]. These conflicting findings potentially imply that any *FTO*-related variations in blood lipids and other CVD risk markers, such as glucose and insulin, may be mediated by the influence of the *FTO* gene on weight [8]. Consequently, to help determine if weight management strategies are warranted, experiments that carefully control for weight and other characteristics are needed to elucidate the influence of the *FTO* per se on blood lipid concentrations. 

No study has assessed how the *FTO* modulates postprandial lipid levels in controlled settings. This is important because atherosclerosis, the major underlying cause of CVD, has been strongly linked to disturbances in postprandial lipoprotein metabolism, and humans in westernized settings spend most of their waking hours (~18 h) in an absorptive state [9,10]. Postprandial TG has been implicated in the development of atherosclerosis, with higher concentrations shown to facilitate the development of atherosclerotic plaques over time [11]. Consistent with this, postprandial TG is a stronger predictor of CVD risk than fasting concentrations [12], although inter-individual variability exists [9]. Thus, it is important to determine if common mutations in the *FTO* influence postprandial lipid responses, especially in individuals with similar characteristics known to affect postprandial TG [9,11].

Our primary aim was to investigate the independent influence of the *FTO* rs9939609 SNP on fasting and postprandial blood lipid concentrations in anthropometrically- and demographically-similar AAs and TTs. Additionally, as a secondary aim, we examined the influence of the *FTO* rs9939609 SNP on fasting and postprandial concentrations of glucose and insulin.

## 2. Materials and Methods 

### 2.1. Participants

This study comprises exploratory endpoints from a study approved by the Loughborough University ethical advisory committee, which was conducted in line with the principles of the Declaration of Helsinki (clinicaltrials.gov identifier: NCT0302537) [13]. Participants were initially recruited through poster advertisement and via word of mouth to a database study where they gave written informed consent. Two hundred and two males of mixed European descent who were aged 18–50 years were recruited. Participants were excluded if they suffered from any medical or psychiatric conditions, had eating disorders or food allergies, and if they smoked. As described elsewhere [13], height, body weight, waist circumference, body fat percentage, and physical activity were measured. A venous blood sample was collected and DNA was extracted using the QIAamp DNA Blood Midi Kit (Qiagen, Manchester, UK) according to the manufacturer’s instructions. Genotyping for the *FTO* rs9939609 SNP was performed using the KBioscience Competitive Allele-Specific Polymerase chain reaction SNP genotyping system by LGC Limited (Hertfordshire, UK). 

After invitation and informed consent to the next phase, a group of 12 AA and 12 TT participants were recruited from our database for a randomized cross-over trial [13]. Participants habitually consumed breakfast on 5 or more days of the week and were weight stable; that is, their weight fluctuated less than 3 kg in the previous 3 months. To attenuate confounding influences, participant groups presented similar anthropometric measures, age, and peak oxygen uptake (Table 1) [13]. 

### 2.2. Preliminary Measures

Participants attended the laboratory for initial preliminary measures. The anthropometric measurements described in the first database visit were repeated and comprised assessments of body weight, height, waist circumference, and body fat percentage. Using methods described previously [14], participants then performed an incremental peak oxygen uptake test on a motorized treadmill.

### 2.3. Experimental Procedure

After the preliminary measures visit, participants completed two trials, control and exercise, in a randomized cross-over design separated by 7–14 days [13]. The primary aim of the present analysis was to examine the influence of the *FTO* rs9939609 SNP per se on blood lipids. Therefore, the subsequent methods and data presented in this manuscript are exclusively from the first 7.5 h of the control trial, to exclude the confounding effects of exercise [15] and ad libitum energy intake [16] on primary endpoints in this analysis. During the control trial, participants rested and consumed a standardized test meal at 2.5 h and an ad libitum buffet meal at 7.5 h. 

In the 24 h before the control trial, participants were instructed not to consume alcohol. They were also told not to engage in strenuous physical activity to preclude any confounding effects of exercise on the study outcomes [15]. A weighed food diary was completed in the same period, and a standardized pizza meal was consumed between 19:00 and 20:00 the night before the control trial to mitigate the effect of prior food intake on fasting and postprandial endpoints [17]. This meal consisted of 1243 kilocalories and the macronutrient content was 52% carbohydrate, 25% fat, and 23% protein.

**Figure 1:**
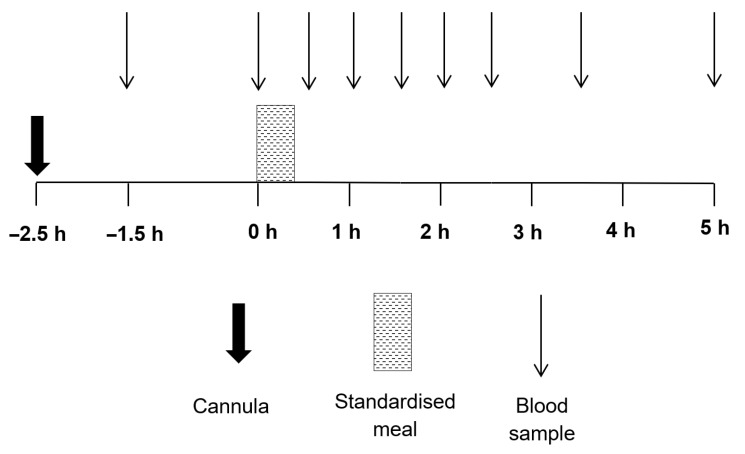
Schematic of trial. Abbreviations: h, hour.

After an overnight fast, participants arrived in the morning of the control trial at approximately 08:30. Participants verbally confirmed adherence to all standardization procedures before the main trial commenced. A cannula was inserted into an antecubital vein 60 min before the baseline blood sample was collected to attenuate any stress response influencing the concentrations of biomarkers (Figure 1) [18]. After the baseline sample (−1.5 h), participants rested for a further 1.5 h before a pre-meal fasting blood sample (0 h) was collected. Participants then consumed a standardized meal consisting of white rolls, butter, cheese, crisps, chocolate slices, and milkshake. The meal contained 1344 kilocalories and the macronutrient composition was 43% carbohydrate, 42% fat, and 15% protein. After, participants rested for 5 h and were able to read, work, and watch TV through laptops and tablet computers.

### 2.4. Blood Sampling

The baseline sample was collected 1.5 h before a pre-meal sample (Figure 1; −1.5 h). A pre-meal blood sample was collected immediately before participants were provided with the standardized meal (0 h). Thereafter, samples were collected every 30 min for 2.5 h, with further samples collected at 3.5 h and 5.0 h. Blood samples were collected into chilled EDTA monovettes (Sarstedt, Leicester, UK) to measure circulating concentrations of TG, non-esterified fatty acids (NEFA), glucose, and insulin. Circulating total cholesterol, HDL, and low-density lipoprotein cholesterol (LDL) were measured solely from baseline (−1.5 h) samples. Samples were centrifuged at 2383× *g* for 10 min at 4 °C. The plasma supernatant was dispensed into a storage tube and then stored at −80 °C until analysis.

### 2.5. Biochemical Analysis

Plasma TG, total cholesterol, HDL, LDL, glucose (HORIBA Medical, Montpellier, France), and NEFA (Randox Laboratories Ltd., County Antrim, UK) concentrations were analyzed using a Pentra 400 benchtop analyzer (HORIBA Medical, Montpellier, France). Plasma insulin concentration was assessed using a commercially available enzyme-linked immunoassay (Mercodia AB, Uppsala, Sweden). Within-batch coefficients of variation for TG, total cholesterol, NEFA, HDL, LDL, glucose, and insulin were 1.7%, 0.7%, 0.5%, 0.9%, 0.9%, 0.6%, and 1.9%, respectively. 

### 2.6. Statistical Analyses

Since the current manuscript presents secondary outcomes, a formal power calculation was not performed and our sample size was equal to that in our primary analysis [13]. Data were analyzed using the IBM SPSS software version 23.0 (IBM Corporation, New York, NY, USA). The homeostasis model assessment of insulin resistance (HOMA-IR) was calculated from baseline (−1.5 h) glucose and insulin concentrations [19]. Time-averaged total area under the curve (TAUC) values from pre-meal (0 h) to five hours after the meal were calculated for TG, glucose, insulin, and NEFAs using the trapezium rule. 

Linear mixed models with genotype (AAs and TTs) as the only fixed factor were used to examine differences in baseline measures (−1.5 h), pre-meal measures (0 h), and time-averaged TAUC responses. Differences in TG, NEFA, glucose and insulin concentrations over time from the pre-meal sample (0 h) and for five hours postprandially were examined with linear mixed models including genotype and time as fixed factors. Absolute standardized effect sizes (ES) were calculated by dividing the difference between the mean values (AAs vs. TTs) with the pooled standard deviation [20]. An ES of 0.2, 0.5, and 0.8 was considered small, moderate, and large, respectively [20]. Mean differences between genotype groups and their respective 95% confidence intervals (CI) were calculated. Statistical significance was accepted as *p* < 0.05. Unless otherwise stated, data presented in text and figures are shown as mean ± standard error of mean (SEM), whereas descriptive data are presented as mean ± standard deviation. 

## 3. Results

### 3.1. Participant Characteristics

No differences were observed between AAs and TTs for age, height, body weight, BMI, waist circumference, body fat%, lean body mass, peak oxygen uptake, or physical activity (*p* ≥ 0.120) (Table 1). Moreover, energy intake in the 24 h prior to the control trial was similar in AAs (2274 ± 142 kcal) and TTs (2302 ± 213 kcal; *p* = 0.716).

### 3.2. Blood Markers

No statistical differences in baseline (−1.5 h) and pre-meal (0 h) concentrations were shown for TG (*p* ≥ 0.293; ES ≤ 0.44), NEFA (*p* ≥ 0.695; ES ≤ 0.16), glucose (*p* ≥ 0.472; ES ≤ 0.30), and insulin (*p* ≥ 0.501; ES ≤ 0.28) between AAs and TTs. There were likewise no statistical differences in HOMA-IR (*p* = 0.625; ES = 0.20) at baseline (−1.5 h), and no differences in baseline (−1.5 h) concentrations of total cholesterol (*p* = 0.964; ES = 0.02), LDL (*p* = 0.725; ES = 0.15), and HDL (*p* = 0.716; ES = 0.15) (Table 2).

**Figure 2:**
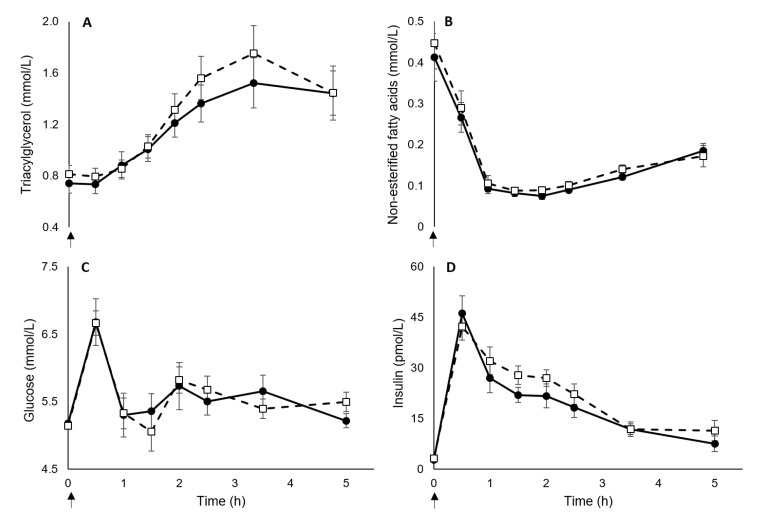
Triacylglycerol (**A**), non-esterified fatty acids (**B**), glucose (**C**), and insulin (**D**) concentrations in AAs (*n* = 12) and TTs (*n* = 12) during the five-hour trial (AAs: Solid lines and filled circles; TTs: dashed lines and unfilled squares). Arrow represents point of standardized meal provision. Data are represented as mean ± standard error of mean (SEM). Abbreviations: h, hour.

Linear mixed models revealed no main effect of *FTO* rs9939609 genotype on postprandial concentrations of TG (mean difference: −0.09 mmol/L; 95% CI −0.43 to 0.25 mmol/L; *p* = 0.576) and NEFA (mean difference: −0.01 mmol/L; 95% CI −0.06 to 0.03 mmol/L; *p* = 0.536) (Figure 2). There were main effects of time for TG and NEFA as concentrations increased and decreased, respectively, in response to meals (*p* < 0.001), but no genotype by time interactions were seen (*p* ≥ 0.545). There was no main effect of *FTO* rs9939609 genotype on concentrations of glucose (mean difference: 0.01 mmol/L; 95% CI −0.40 to 0.41 mmol/L; *p* = 0.976) and insulin (mean difference: −2.69 pmol/L; 95% CI −7.67 to 2.29 pmol/L; *p* = 0.274) during the trial. Main effects of time were identified for glucose and insulin concentrations (*p* < 0.001), although there were no genotype by time interactions for either outcome (*p* ≥ 0.586).

**Figure 3:**
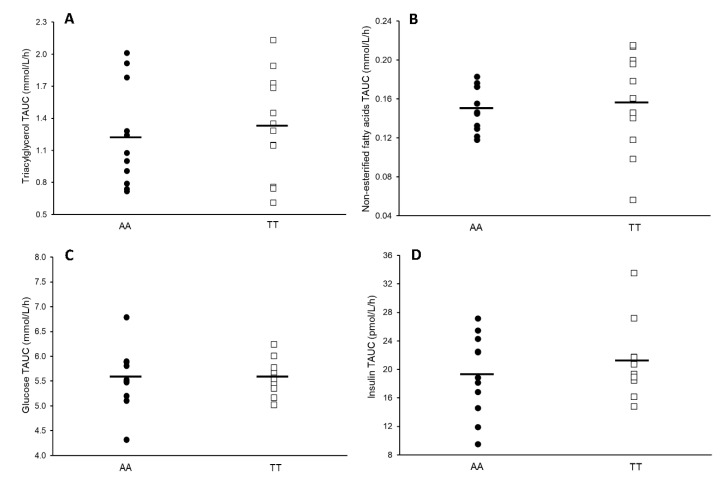
Time-averaged total area under the curve (TAUC) values for triacylglycerol (**A**), non-esterified fatty acids (**B**), glucose (**C**), and insulin (**D**) in AAs (*n* = 12) and TTs (*n* = 12) (AAs: filled circles; TTs: unfilled squares). Data points represent individual participant values and the black line represents the mean. Abbreviations: TAUC, total area under the curve.

No statistical between-genotype differences in time-averaged TG TAUC (mean difference: −0.10 mmol/L/h; 95% CI −0.52 to 0.31 mmol/L/h; *p* = 0.606; ES = 0.22) and NEFA TAUC (mean difference: −0.01 mmol/L/h; 95% CI −0.04 to 0.03 mmol/L/h; *p* = 0.734; ES = 0.15) were seen (Figure 3). Genotype groups also showed statistically similar TAUC values for glucose (*p* = 0.951; ES = 0.03) and insulin (*p* = 0.395; ES = 0.37). 

### 3.3. Retrospective Power Calculations

Given the exploratory nature of this analysis, retrospective power calculations using G*Power [21] were undertaken to discern the required power, sample size, and effect size for all fasting (baseline) and postprandial (time-averaged TAUC) blood lipid, glucose, and insulin outcomes presented. Using the standardized effect sizes (Cohen’s d) calculated in this analysis, a post hoc power calculation revealed that the current analysis with 12 AAs and 12 TTs had between 5.0 and 17.8% power to detect between-genotype differences in all study outcomes. Furthermore, it was estimated that a sample size between 166 (83 per group; fasting TG) and 91,894 (45,947 per group; fasting total cholesterol) would be required to detect the reported effect sizes (d) for each study outcome (alpha: 0.05; 1–β: 0.8; group allocation: 1:1). In the present analysis of 12 AAs and 12 TTs, an effect size (d) of 1.20 would be required to detect between genotype differences at the 0.05 level and with 80% power. Therefore, the minimum absolute difference between AAs and TTs that could be detected with an alpha value of 0.05 for the postprandial outcomes in this analysis is: TG 0.57 mmol/L/h, NEFA 0.05 mmol/L/h, glucose 0.59 mmol/L/h, and insulin 6.53 pmol/L/h.

## 4. Discussion

The present study indicates that postprandial concentrations of TG, NEFA, glucose, and insulin were similar in a male cohort of *FTO* rs9939609 AAs and TTs. Fasting concentrations of TG, NEFA, total cholesterol, LDL, HDL, glucose, insulin, and HOMA-IR were also similar between AAs and TTs.

Carriers of the *FTO* rs9939609 risk allele have a higher risk of CVD than non-carriers [5,6], but it is not clear if blood lipids, particularly in the postprandial state, mediate this association. While most studies examining the effect of the *FTO* obesity SNPs on blood lipids have assessed fasting concentrations [8,22], higher postprandial TG concentrations are crucial in the development of atherosclerotic plaques via oxidative stress and display stronger relationships with CVD than fasting levels [12]. One study reported that random TG concentrations were, on average, 0.16 mmol/L higher in AAs than TTs. However, this study had confounding influences, namely the lack of control over nutritional state, and the fact that AAs presented a higher BMI. Our study mitigated these confounding effects and showed that postprandial TG concentrations are similar between AAs and TTs, indicating that the *FTO* rs9939609 SNP per se does not affect postprandial variations in TG. Furthermore, in line with previous evidence [6,8], we observed no influence of the *FTO* rs9939609 SNP on fasting TG concentrations. Previous studies indicate that a 1 mmol/L rise in TG increases CVD risk by 14% in males [23]; thus, the small 0.09–0.10 mmol/L differences in TG concentrations throughout the trial between AAs and TTs is likely to exert a negligible influence on CVD risk. Further studies are needed to elucidate other genetic and non-genetic factors that affect TG because large heterogeneity in concentrations are unaccounted for [9].

High concentrations of NEFA are a risk factor for elevated CVD, possibly through the pathological progression of atherosclerosis with other blood lipids [24]. The present study suggests that the *FTO* rs9939609 SNP does not influence fasting and postprandial NEFA concentrations. Moreover, we saw no influence of the *FTO* rs9939609 SNP on fasting total cholesterol, LDL, and HDL at baseline (−1.5 h), with trivial ESs shown. This supports work that has compared cholesterol concentrations between *FTO* genotypes and accounted for body weight through adjustments in statistical analyses [8].

The *FTO* SNPs within intron one are the most penetrant common polymorphisms related to obesity in Europeans [2]. This makes it challenging to examine the intrinsic influence of these genetic regions on CVD-related endpoints, because of the link between obesity and CVD [9]. Further, it may partly explain the mixed findings reported in studies that have assessed *FTO*-related differences in blood lipids and other CVD markers, including glucose, insulin, blood pressure, and inflammatory measures [5,6,7,8]. Since we compared relatively homogenous individuals who were free from metabolic conditions, and we enforced strict meal and physical activity standardization procedures, the present findings suggest that blood lipids are not affected by the *FTO* rs9939609 SNP per se. Rather, our data implies that less favorable blood lipid levels in individuals carrying the *FTO* risk allele versus non-carriers may be mediated by the higher adiposity displayed in these individuals. Accordingly, efficacious prevention or treatment of *FTO*-related differences in blood lipids is likely to require obesity management strategies. Such strategies should include satiety control, given the *FTO* risk allele increases appetite and food intake [25]. Increasing engagement in physical activity should also be recommended, as evidence suggests that higher activity levels may offset deleterious metabolic consequences of the *FTO* gene [26].

Our previous analyses of this cohort demonstrated that individuals with the *FTO* rs9939609 AA genotype exhibited higher postprandial levels of acylated ghrelin compared to TTs when resting [13]. Ghrelin has been implicated in glucose homeostasis, with higher levels shown to prevent hypoglycemia [27] and lower insulin release [28]. As a result, it could be postulated that glucose and insulin levels may differ between AAs and TTs. We found, however, that fasting and postprandial concentrations of glucose and insulin were similar in AAs and TTs. Previous work has reported higher fasting and postprandial glucose and insulin concentrations in carriers of obesity-risk *FTO* allele, yet this is eradicated when adjusted for BMI [8]. In this context, findings from the current study are consonant with evidence suggesting that *FTO* obesity SNPs per se do not influence parameters of insulin resistance. 

Our results are limited to mixed European males and hence additional studies are required to verify our findings in females and other racial groups, including Blacks and Asians. Additional studies are also needed in individuals with dyslipidemia and CVD, though it is notable that we sought to recruit metabolically healthy individuals to decrease the bias and noise associated with studying individuals with metabolic conditions [29]. Another limitation is that we only assessed one SNP, yet we note that the rs9939609 SNP is the most commonly examined *FTO* SNP and is in high linkage disequilibrium with other SNPs related to obesity and metabolic disease [2]. A further limitation is that this analysis was exploratory, and a relatively small sample size was recruited. However, our effect sizes were small or negligible and AAs and TTs with comparable demographic and anthropometric traits were tested in controlled, standardized conditions, improving the ability to discern the independent influence of the *FTO* rs9939609 SNP on blood lipids. It is also noteworthy that we have identified appetite-related hormone differences between AAs and TTs in this cohort [13]. For these reasons, we believe our findings indicate that the *FTO* rs9939609 SNP exerted minimal influence on the exploratory outcomes in this analysis; nevertheless, studies with larger samples are needed to confirm this hypothesis.

## 5. Conclusions

In conclusion, in individuals with similar demographic characteristics and adiposity, the *FTO* rs9939609 SNP did not influence fasting and postprandial blood lipids, glucose, and insulin. Although larger studies in other populations are needed, it is likely that adverse blood lipid profiles in carriers of the *FTO* rs9939609 SNP A-allele are mediated by adiposity, suggesting obesity prevention and treatment strategies should be paramount in improving the blood lipid profiles of *FTO* risk allele carriers.

## Figures and Tables

**Table 1:** Descriptive characteristics of the AAs and TTs.

	AA (*n* =12)	TT (*n* = 12)	TT vs. AA Mean Difference (95% CI ^1^)
Age (year)	20.9 ± 3.5	21.3 ± 3.6	−0.4 (−3.4 to 2.6)
Height (cm)	181.6 ± 5.8	177.5 ± 6.5	4.1 (−1.2 to 9.3)
Weight (kg)	77.6 ± 11.3	73.8 ± 6.9	3.9 (−4.1 to 11.8)
Body mass index (kg/m^2^)	23.5 ± 2.7	23.5 ± 2.3	0.0 (−2.1 to 2.1)
Waist circumference (cm)	80.3 ± 6.1	78.1 ± 4.1	2.2 (−2.2 to 6.6)
Body fat percentage (%)	15.6 ± 5.1	13.9 ± 4.7	1.7 (−2.4 to 5.9)
Lean body mass (kg)	65.2 ± 7.4	63.3 ± 4.2	1.9 (−3.2 to 7.0)
Peak oxygen uptake (mL/kg/minute)	55.8 ± 5.8	56.6 ± 4.9	−0.8 (−5.4 to 3.7)
Physical activity (metabolic equivalent minutes/week)	4368 ± 1968	4790 ± 2728	−423 (−2436 to 1591)

Values are mean ± standard deviation. Data were analyzed using linear mixed models with genotype (AA or TT) included as a fixed factor. No main influence of genotype was identified (*p* ≥ 0.120). ^1^ 95% CI of the mean absolute difference between genotype groups. Abbreviations: CI, confidence interval.

**Table 2:** Fasting plasma concentrations and HOMA-IR at baseline (−1.5 h) and pre-meal (0 h) in AAs and TTs.

	AA (*n* = 12)	TT (*n* = 12)	TT Vs. AA Mean Difference (95% CI ^1^)	Effect Size
Triacylglycerol (mmol/L)				
Baseline	0.77 ± 0.08	0.88 ± 0.07	−0.11 (−0.32 to 0.10)	0.44
Pre-meal	0.74 ± 0.08	0.81 ± 0.07	−0.07 (−0.28 to 0.14)	0.28
Non-esterified fatty acids (mmol/L)				
Baseline	0.46 ± 0.09	0.43 ± 0.06	0.03 (−0.18 to 0.25)	0.12
Pre-meal	0.41 ± 0.06	0.45 ± 0.06	−0.03 (−0.21 to 0.14)	0.16
Total cholesterol (mmol/L)				
Baseline	3.40 ± 0.12	3.41 ± 0.18	−0.01 (−0.47 to 0.45)	0.02
Low-density lipoprotein cholesterol (mmol/L)				
Baseline	1.77 ± 0.12	1.83 ± 0.14	−0.07 (−0.45 to 0.32)	0.15
High-density lipoprotein cholesterol (mmol/L)				
Baseline	1.28 ± 0.07	1.24 ± 0.11	0.05 (−0.22 to 0.31)	0.15
Glucose (mmol/L)				
Baseline	5.35 ± 0.11	5.26 ± 0.06	0.09 (−0.17 to 0.35)	0.30
Pre-meal	5.18 ± 0.08	5.15 ± 0.04	0.03 (−0.16 to 0.23)	0.14
Insulin (pmol/L)				
Baseline	3.34 ± 0.47	3.09 ± 0.52	0.25 (−1.21 to 1.72)	0.15
Pre-meal	2.82 ± 0.34	3.26 ± 0.55	−0.44 (−1.76 to 0.89)	0.28
HOMA-IR				
Baseline	0.81 ± 0.12	0.72 ± 0.12	0.08 (−0.27 to 0.44)	0.20

Values are mean ± standard error of mean. Data were analyzed using linear mixed models with genotype (AA or TT) included as a fixed factor. No main influence of genotype was identified (*p* ≥ 0.293). ^1^ 95% CI of the mean absolute difference between genotype groups. Abbreviations: CI, confidence interval; HOMA-IR, Homeostatic model assessment of insulin resistance; h, hours.
